# Supplementary material for: Causal Relationship between Aging and Anorexia Nervosa: A White-Matter-Microstructure-Mediated Mendelian Randomization Analysis
Source: Biomedicines. 2024 Aug 16;12(8):1874. doi: 10.3390/biomedicines12081874 (PMC11351342; doi:10.3390/biomedicines12081874)
Supplement: Supplementary file 1 [file biomedicines-12-01874-s001.zip › Supplementary Figure/Figure S8/Leaveoneout plot of MR analysis for TL to ubm-b-2045.pdf]

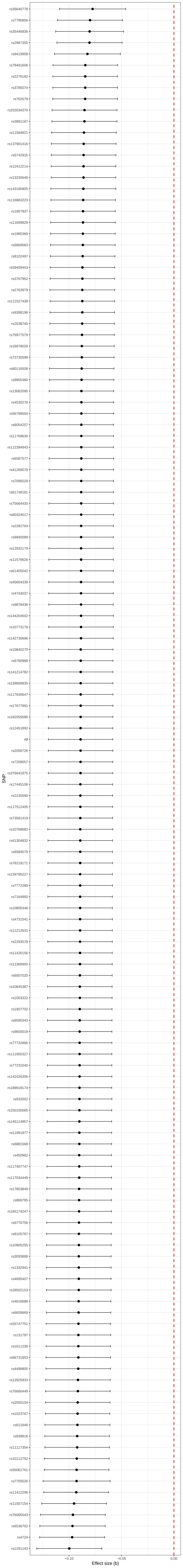

rs35640778
rs7790856
rs35446936
rs2987355
rs9419958
rs78491606
rs2276182
rs3785074
rs762679
rs202034370
rs3891167
rs11584821
rs137901416
rs5742915
rs12412214
rs13230646
rs143190905
rs116863223
rs1957937
rs11699829
rs1985369
rs6669563
rs8102497
rs59409453
rs3767952
rs2763979
rs111527438
rs9398196
rs2538745
rs79977579
rs16978028
rs73730598
rs80116508
rs9955360
rs13062095
rs4530278
rs56799554
rs6054257
rs11769630
rs112394943
rs6587577
rs41269079
rs7099229
rs61748181
rs75664430
rs80324517
rs2282764
rs9940099
rs12932179
rs11579626
rs61405042
rs45604339
rs4743037
rs9878436
rs144204502
rs10773176
rs142730696
rs10840270
rs6790988
rs141214782
rs139669835
rs117630647
rs17677991
rs182059586
rs12451892
All
rs2056726
rs7209057
rs376641875
rs17445108
rs2230590
rs117512405
rs73581419
rs10768683
rs41304832
rs6584579
rs76219171
rs139795227
rs7772289
rs7164950
rs10805346
rs4731541
rs11212631
rs2293579
rs11426156
rs12369950
rs6007020
rs10845387
rs1003322
rs1907702
rs6590343
rs9600019
rs77732866
rs111950327
rs77231040
rs142426306
rs188918174
rs932002
rs150150565
rs145114957
rs11991877
rs6881568
rs450962
rs117407747
rs117034449
rs17803849
rs869785
rs185174247
rs6776756
rs8105767
rs10905255
rs3093888
rs1332941
rs4605407
rs28502153
rs4616688
rs6659669
rs55747751
rs131797
rs1611236
rs66731853
rs4498805
rs12925933
rs76666449
rs2555104
rs1023767
rs611646
rs939916
rs11117354
rs10112752
rs56061761
rs7705526
rs11412296
rs11557154
rs76065543
rs6536702
rs4724
rs1291143
SNP
-0.10
-0.05
0.00
Effect size (b)
